# Evaluation of the Development Potential of Hainan Free Trade Port Industrial Chain Driven by the Internet of Things Distribution Mechanism

**DOI:** 10.1155/2022/8146926

**Published:** 2022-04-30

**Authors:** Jing Yang, Xiaoying Zhang, Xiuying Yang

**Affiliations:** ^1^School of Information Engineering, Hainan Vocational University of Science and Technology, Haikou, Hainan 571126, China; ^2^School of Finance, Hainan Vocational University of Science and Technology, Haikou, Hainan 571126, China

## Abstract

This paper starts from the industrial structure and characteristics of the Internet of Things and compares and analyzes the development ideas of the Internet of Things industry in the market economy under different systems. We also discuss IoT business model innovations and analyze the inadequacies of current IoT business models. We use the cost-benefit analysis method to analyze, from the aspects of social benefits, economic benefits, environmental benefits, and externalities. In this paper, factor analysis and quantitative statistics are used to calculate the weight of influencing factors, and the multilevel fuzzy evaluation method is used to score the influencing factor indicators to calculate the final score result, so as to judge the applicability of the multilevel fuzzy evaluation method to judge the profit model. In order to verify whether the establishment of economic regions plays a positive role in regional economic development, a double-difference model is established. Two sets of data from the Free Trade Zone and Hainan Free Trade Zone are selected for regression analysis, and robustness tests of various methods are carried out. This paper regresses various samples of the Hainan Free Trade Zone by region and city level to explore their characteristics in different regions. From the empirical results, it can be seen that the establishment of the Hainan Free Trade Zone has indeed promoted the economic development of the region where it is located, and this result is still significant after repeated robustness tests. Judging from the regression results by region, the establishment of the Hainan Free Trade Zone has a greater role in promoting the economic development of the eastern and western regions and it has a stronger pulling effect on the western regions than in the western regions. The policy effect of establishing a free trade zone may not be significant due to too few samples and short establishment time.

## 1. Introduction

The diversification of network access, IP broadband, and computer software technology has developed rapidly, and the ability to collect and classify processing based on massive information has been greatly improved [1, 2]. The development of the Internet of Things is to further deepen the application of information technology and gradually realize the automation of information collection, which is bound to be a “radical innovation” in the development of information technology. The huge market potential and development prospects of the Internet of Things have attracted great attention from governments, scientific research institutions, and related enterprises in various countries [3].

At present, the application of the Internet of Things can be mainly divided into several major fields: family life, product logistics, intelligent transportation, public transportation, public safety, smart campus, medical care, and intelligent business [4]. In recent years, some more mature IoT solutions have been introduced one after another. For example, IBM Sweden's smart transportation solution is a relatively mature application, which can predict traffic demand by collecting real-time traffic data and identifying traffic flow and vehicle usage patterns. It can improve the point-to-point traffic experience, improve the operational efficiency of the transportation system, reduce environmental impact, and ensure the safety and confidentiality of the implementation plan. With the smart transportation solutions, IBM has brought huge benefits to the Swedish city of Stockholm and is a global model for smart transportation. In recent years, the development data of the Internet of Things has confirmed its huge commercial value and broad market prospects to the world [5].

At this stage, the theoretical research on the operation of the Internet of Things is still relatively small, and it has not yet risen to the theoretical level. Therefore, it is necessary to introduce the two-sided market theory into the theoretical research of the Internet of Things, discuss it by building a two-sided market model of software service providers-operators-end users, and use the method of numerical simulation to study the two-sided pricing structure and user scale of IoT platform enterprises. This paper discusses the construction of the IoT industry chain from the perspective of the two-sided market theory, which helps to better understand the pricing and competition strategies of the IoT platform companies and the government's management of platform companies and provides references for subsequent research on the IoT industry chain. This paper comprehensively applies industry chain theory, game theory, and two-sided market theory to the research field of the industry chain, reflecting the cross-integration of disciplines. This will help all entities in the industry chain to correctly understand and grasp the operation mechanism of the Internet of Things led by operators, provide opinions and references for relevant enterprises in the industry chain to improve the business model of the Internet of Things, and help enterprises identify the impact on the development of the Internet of Things industrialization. The key influencing factors in the industry are as follows: timely identifying opportunities, responding to risks, and adapting to the market environment as soon as possible. This paper focuses on the research on the bilateral pricing structure of IoT enterprise platforms, which will serve as a reference for Chinese operators to formulate their future development strategies. At the same time, it provides theoretical guidance and experience reference for relevant government departments to guide and plan the demonstration application of the Internet of Things industry in the future and improve market service capabilities.

By comparing and analyzing the path modes of different countries to develop the Internet of Things economy, through the systematic analysis and exposition of the development and operation trajectories of the Internet of Things industries in developed countries, the current situation of the international Internet of Things industry development is deeply interpreted. This paper adopts the multilevel fuzzy evaluation method to comprehensively evaluate the profit model, reflects the applicability of the profit model through the evaluation results, and analyzes the possibility of long-term development of the profit model. The main research methods of this paper are the comparative research method and the empirical analysis method. The comparative research method means that the Hainan Free Trade Bonded Area and the Free Trade Zone are always compared in the writing and topic selection of this article. From the development status to their respective preferential tax policies, as well as subsequent empirical analysis, the two special economic regions are compared to analyze the similarities and differences between the two special economic regions that promote opening to the outside world. The empirical analysis method means that this paper establishes a model and uses two sets of data from the Hainan Free Trade Bonded Area and the Free Trade Zone to verify whether the establishment of the Hainan Free Trade Bonded Area and the Free Trade Zone has played a positive role in the economy of the city and province where it is located.

## 2. Related Work

Based on the driving model of IoT demand, relevant scholars divide the development of IoT into two stages: the “visible hand” (government) driving stage and the “invisible hand” (market) driving stage [6]. The researchers believe that in the visible hand-driven stage, the core appeal of IoT business model innovation lies in how to strengthen the administrative efficiency of social management of public management agencies and solve the cost and profit problems of economic management. The core appeal of the networked business model is how to solve the problems of low-cost operation and profit growth of major economic organizations. Relevant scholars believe that there are two paths in the innovation of IoT business models, namely, the core of developer application and the core of conquering high-end technology [7]. There are two types of IoT business models for telecom operators to innovate and expand the industrial chain: strong cooperation and omnipotent platform. Therefore, whether it is the business practice of operators or the theoretical discussion of scholars, it is pointed out that the lack of a mature business model is the key factor restricting the development of the industrialization of the Internet of Things.

Relevant scholars believe that the development of the Internet of Things industry should pay attention to the innovation of business models and put forward feasible business models based on the wide application of the Internet of Things: the government-paid model, the free model, the operator-driven model, the user-manufacturer joint promotion model, the vertical application model, and the industry common platform model [8]. Based on the life cycle of IoT products, relevant scholars have studied the business model of the Internet of Things for Chinese operators according to the introduction period, growth period, and maturity period [9]. The researcher's study the business model of the Internet of Things from the perspective of operators and believes that the business model of the Internet of Things dominated by operators includes cooperative development, independent promotion, independent development and promotion, and customer customization [10]. Relevant scholars believe that the future business models of IoT development mainly include the government BOT model, channel and cooperation model, advertising model, and self-operated model [11]. Relevant scholars divide the business model of the Internet of Things into a customer-built model, a platform leasing operation model, an advertising model, a government BOT model, and a mobile payment model [12]. In addition, relevant scholars analyzed the development status of China Telecom in the field of the Internet of Things and pointed out that the biggest bottleneck in the development of the Internet of Things at this stage is the lack of a win-win cooperation model and a mechanism for mutual trust in the whole society [13].

From the perspective of project management, the management and operation mode of the construction of the Internet of Things technology park is analyzed. Relevant scholars believe that the brand strategy, business model, and technological innovation of the industrial park can help expand the overall influence of the Internet of Things industrial cluster and drive the development of related industries [14]. The researchers believe that industrial clusters based on the enterprise network structure are helpful for vertical knowledge spillover, horizontal knowledge integration, and breakthrough of core technologies, thus forming a benign cluster culture that encourages cooperation and innovation [15]. Based on the perspective of the global value chain, relevant scholars combined the new characteristics and development trends of modern industrial-organizational structure agglomeration, integration, and modularization, from the aspects of the dynamic mechanism, innovation mechanism, benefit mechanism, industry-university-research collaboration mechanism, and development and evolution mechanism [16–18]. The Internet of Things industry cluster in the Yangtze River Delta region conducts systematic research and puts forward relevant suggestions. Based on the agglomeration factor model and logistic growth model, relevant scholars believe that regional resource endowment, market, government regulation, industrial environment, and specific knowledge are the main factors affecting the IoT industry cluster [19, 20].

The impact of the researchers on the regional economy is divided into the impact on employment, on the introduction of related industries, on the regional logistics strength, and on the regional finance [21]. Relevant scholars take Beijing Tianzhu Comprehensive Hainan Free Trade Zone as the research object to study its role in Beijing's economic development and point out that Beijing Tianzhu Comprehensive Hainan Free Trade Zone has played a driving role in two aspects: promoting the upgrading of processing trade and stimulating scientific and technological services [22, 23].

## 3. Methods

### 3.1. Analysis of Internet of Things Industry Composition

The IoT industry chain is divided into three parts: upper, middle, and lower. The upstream part is composed of communication suppliers, chip suppliers, external hardware suppliers, radio frequency identification, and sensor suppliers; the midstream part includes system equipment manufacturers, system integrators, system platforms, and software integrators; and the downstream part consists of telecom operators and IoT operators.

The potential market scale of the Internet of Things industry is huge, and the development space along with the innovation of Internet of Things technology will continue to bring benefits from huge industrial clusters. The industrial cluster effect of the Internet of Things has changed government investment, and the government's “point-to-point” investment form has changed to the current “point-to-chain” investment method, that is, adopting the method of cluster investors and trying to invest in enterprises that cover certain clusters in the IoT industry chain.

**Figure 1:**
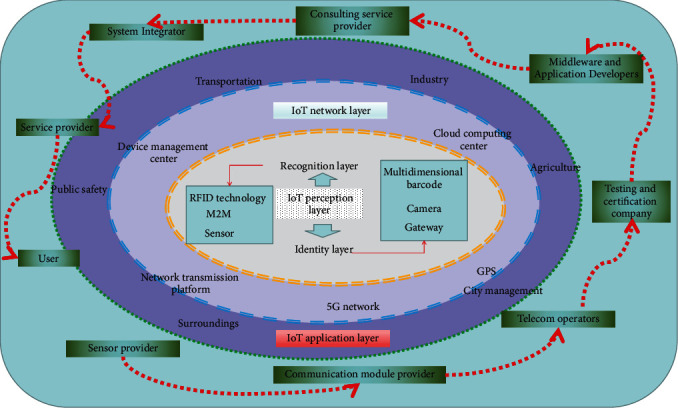
Schematic diagram of the composition of the IoT industry chain.

Although each enterprise is one of the enterprises in the industrial chain of the Internet of Things, there is still no good relationship of mutual cooperation. The maximum benefit can be achieved only by integrating government resources. Otherwise, these Internet of Things enterprises are still small- and medium-sized enterprises. Without strong capital and a strong technical team, business investment is prone to bottlenecks and is difficult to overcome. Only the government as the link of its cooperation can effectively integrate its resources. The schematic diagram of the Internet of Things industrial chain is shown in Figure 1.

The role of a good communication platform is to closely link the upper, middle, and downstream industries of the IoT industry and to use it as the core to realize the vertical linkage of the IoT industry and promote the coordinated development of the IoT industry chain. Regardless of the advancement of any link in the IoT industry chain, it will drive the simultaneous development of a series of internal industries, organically connect the various branches of the IoT industry, and promote each other to form a virtuous circle, thus bringing about a huge IoT industry cluster.

### 3.2. Analysis of Industrial Effect of the Internet of Things

The economic changes brought about by the Internet of Things can be summarized as follows: (1) It allows all parties involved in the transaction to obtain information in a more timely and accurate manner in the process of business operations. (2) It can reduce the cost of each link in the transaction process, which reduces the financial burden of enterprises. (3) The Internet of Things can make the entire transaction process transparent, and all the data generated are retained on the online transaction platform, allowing the government to grasp it more accurately.

**Figure 2:**
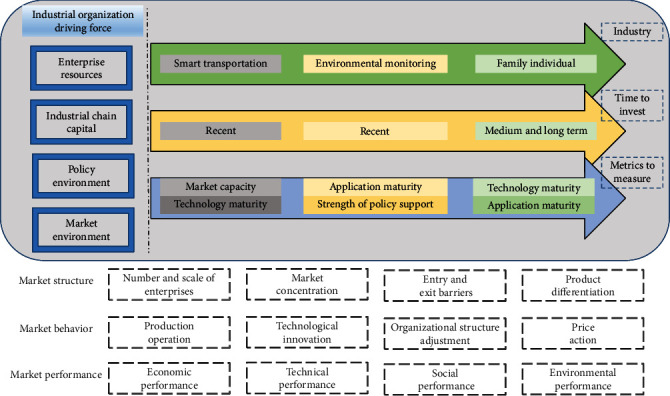
Composition of investment opportunities in the IoT industry applications.

The development of the IoT business model is divided into two levels: one is the government-led promotion stage. The proposal for the Internet of Things has been promoted by the government from the beginning because of the maturity of its technology. One of the key applications is in the field of long-term social development and people's livelihoods, for example, in transportation, environmental protection, urban management, and industry supervision. Under the promotion of the government in China, we establish a nationwide IoT application demonstration application area or IoT development base. At the same time, through this opportunity, a group of enterprises will be supported to develop the IoT industry and the local new economic growth point will be driven, which can effectively and long-term stimulate domestic demand. The second is the market mechanism-driven stage. After the government vigorously promoted the Internet of Things industry and gave corresponding policies, the economic and social management models in the market economy have changed. As a result, the society will have a large demand for Internet of Things applications, so that Internet of Things applications will enter the market. Therefore, considering how to turn the demand point of the Internet of Things into a profitable business model, enterprises in the application layer of the Internet of Things have been actively exploring business models that adapt to various industries. For example, in the exploration of business models, enterprises should first analyze clearly the elements contained in the business model, namely, management and technical teams, market capacity and analysis, accurate positioning of technologies and products, product market development and development plans, financial profit planning and prediction. The composition of investment opportunities for IoT industry applications is shown in Figure 2.

### 3.3. Multilevel Fuzzy Evaluation Method to Judge the Profit Model

When solving practical problems, the situation is usually more complicated and the influencing factors are often multi-faceted. If you want to make an objective and true evaluation, you need to consider the problem more comprehensively. Too many influencing factors, on the one hand, will make it difficult to determine the weight, and on the other hand, the weight value will be too small, making it difficult to distinguish the evaluation results.

Multilevel fuzzy evaluation is an improved method of single-level fuzzy comprehensive evaluation method based on the fuzzy mathematics principle. First, classify the influencing factors, consider the big aspects first and then the small aspects, list the influencing factor indicators from big to small, and then judge the weight of the influencing factors from small to large. The qualitative index is quantified, and finally, the evaluation result is calculated using the fuzzy mathematical synthesis operator formula. The general steps of the multilevel fuzzy evaluation algorithm are as follows:

(1) On the basis of classifying the influencing factors, the influencing factors are regarded as the evaluation objects and the grades are divided according to the evaluation criteria:
  Let *U* represent the set of evaluation criteria and ui represent each evaluation index standard, and arrange them in order from high to low, where *i* = 1, 2, ..., *m*, determine the evaluation standard set *U*.
(2) According to the classification of influencing factors, each influencing factor represents an evaluation index, and a weight value is assigned to each level of the index.
  Let V represent the weight set of the first-level indicators, Vi the weight set of the second-level indicators, *i*_*v*_ the weight of each indicator in the first level, and *v*_*ij*_ the weight of each indicator in the second level. The weight of the first-level indicator is expressed as
    (1) $$\begin{array}{c} \left\{\begin{array}{c} V=\left(v_{1}\;v_{2}\;\cdots \;v_{m}\right)\;v_{i}\in \left(-1,1\right),\\ \prod_{i=1}^{m}\left(v_{i}•v_{i+1}\right)=1. \end{array}\right. \end{array}$$
  The weight of the secondary indicator is expressed as
    (2) $$\begin{array}{c} \left\{\begin{array}{c} V_{i}=\left(v_{i1}\;v_{i2}\;\cdots \;v_{ij}\right)\;v_{ij}\in \left(-1,1\right),\\ \prod_{j=1}^{n}\left(v_{ij}•v_{ij+1}\right)=1. \end{array}\right. \end{array}$$
(3) According to the division of the rating criteria, the fuzzy relationship corresponding to each evaluation index is found, and the evaluation matrix is established.
  Let *R* represent the fuzzy judgment matrix and *r*_*ij*_ represent the membership degree of the ith index in the jth level evaluation standard, and determine the fuzzy judgment matrix.
(4) Use the fuzzy matrix synthesis formula to calculate the comprehensive evaluation result after fuzzy transformation.
  Let *A*_*i*_ represent the second-level fuzzy judgment result matrix, *a*_*ij*_ represent the fuzzy evaluation result corresponding to each evaluation index in the second-level evaluation index, *B*_*i*_ represent the first-level fuzzy evaluation result matrix, and *B* represent the final comprehensive evaluation result.
  The calculation formula of the fuzzy synthesis algorithm is
    (3) $$\begin{array}{c} a_{ij}=\prod_{i=1}^{m}\left[r_{ij}v_{ij}•r_{\left(i+1\right)j}v_{\left(i+1\right)j}\right]. \end{array}$$
  The evaluation results of the secondary indicators are
    (4) $$\begin{array}{c} A_{i}=\left(\begin{array}{cccc} v_{i1} & v_{i2} & \cdots & v_{ij} \end{array}\right)•R=\left(\begin{array}{cccc} v_{i1} & v_{i2} & \cdots & v_{ij} \end{array}\right)•\left[\begin{array}{c} a_{i1}\\ a_{i2}\\ \vdots \\ a_{ij} \end{array}\right]. \end{array}$$
  The first-level index evaluation results are as follows:
    (5) $$\begin{array}{c} B_{i}=A_{i}•\left(\begin{array}{cccc} v_{i1} & v_{i2} & \cdots & v_{ij} \end{array}\right). \end{array}$$
(5) According to the principle of maximum membership degree, it can be known that the value of *B* is the maximum value in *B*_*i*_.

### 3.4. Methods for Judging the Weight of Indicators

To use factor analysis to calculate the weights of indicators, the first step is to test the sample data. Only data that meet the criteria can be used for factor analysis. The number of samples should be at least 5 times the number of variables, and the total number of samples should not be less than 100. Variables that are independent of each other cannot perform dimensionality reduction operations. We perform KMO and Bartlett sphericity tests on the variables that meet the requirements. The KMO value is between 0 and 1, and the KMO value is greater than 0.6 before factor analysis.

Standard processing of the original variable sample data is as follows:

(6) $$\begin{array}{c} x_{j}=\frac{1}{n-1}\prod_{i=0}^{n-1}\left[x_{ij}x_{\left(i+1\right)j}\right],\\ S_{j}^{2}=\frac{1}{n-1}\prod_{i=0}^{n-1}{\left(x_{j}-x_{ij}\right)}^{2}. \end{array}$$

Let *r*_*ij*_ denote the correlation coefficient between the original variables *x*_*i*_ and *x*_*j*_:

(7) $$\begin{array}{c} r_{ij}=\frac{\prod_{k=0}^{n-1}\left(x_{kj}-x_{j}\right)\left(x_{ki}-x_{i}\right)}{\sqrt{\prod_{k=0}^{n-1}{\left[{\left(x_{kj}-x_{j}\right)}^{2}\left(x_{ki}-x_{i}\right)\right]}^{2}}}. \end{array}$$

Calculate the principal component contribution rate *f*_*a*_ and the cumulative contribution rate *f*, respectively. The calculation formula of the contribution rate and the calculation formula of the cumulative contribution rate are

(8) $$\begin{array}{c} f_{a}=\frac{\left(1-\lambda_{i}\right)}{\prod_{k=0}^{n-1}\lambda_{k}},\\ f=\frac{\prod_{k=0}^{n-1}\lambda_{k}}{1+\prod_{k=0}^{m-1}\lambda_{k}}. \end{array}$$

## 4. Results and Analysis

### 4.1. Variable Descriptive Statistics

The establishment of various types of Hainan Free Trade Zones and special economic zones such as free trade zones is different from the implementation of other policies. Whether it is the earliest established Hainan Free Trade Zones or the free trade zones established in recent years, they are not the same group. The establishment is completed, but the establishment is approved by the State Council one after another. Therefore, it is impossible to divide all experimental group and control group samples with a certain policy implementation year as a predetermined standard.

In order to create an important distinction standard between the experimental group and the control group, dummy variables are introduced: bonded area and Free Trade Zone. For the Hainan Free Trade Bonded Zone Group, cities that have established various types of Hainan Free Trade Bonded Zones are marked as 1 and cities that have not established Hainan Free Trade Bonded Zones are marked as 0. For the Hainan Free Trade Port, the province with a free trade zone is marked as 1 and the province without a free trade zone is marked as 0. Thus, in all samples, established and unestablished cities and provinces form a natural comparison.

Regional economic development not only depends on inherent economic factors such as population, educational development, and industrial structure, but the policy effects of important policies will also change the economic development status of the policy implementation areas. Compared with the single-difference method, the double-difference method establishes an experimental group and a control group, uses the main research variables “establishing Hainan Free Trade Zone” and “establishing a free trade zone” as dummy variables, and uses other variables that affect the regional economy as control variables.

Since regional economic development also depends on the resource endowment of each city, such as population, finance, and education, this paper also introduces other variables that affect economic development. Education affects the talent training and personnel quality of a city and will inevitably affect the level of urban economic development.

Different cities have different industrial bases due to historical and technological development, geographical location, and other reasons. For example, historical reasons have shaped the old industrial base in the Northeast, and the unique coastline has enabled Shanghai, which has developed foreign trade, to develop a port economy early. During the planned economy period, the old industrial bases in Northeast China relied on the industry-led industrial structure to achieve the strong development of the regional economy. However, the current industrial upgrading in Northeast China is hindered, resulting in economic decline. Different industrial structures play different roles in regional economic development in different periods. The variable descriptive statistics of the Hainan Free Trade Port are shown in Table 1.

### 4.2. Impact of the Hainan Free Trade Zone on the Regional Economy

**Figure 3:**
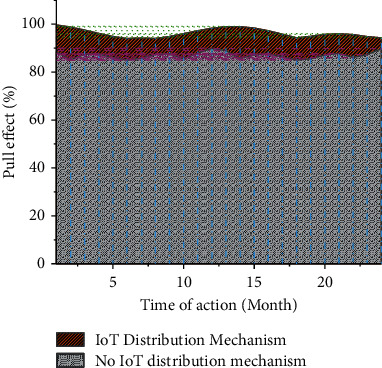
Stimulating effect of the Hainan Free Trade Zone on regional economy.

**Figure 4:**
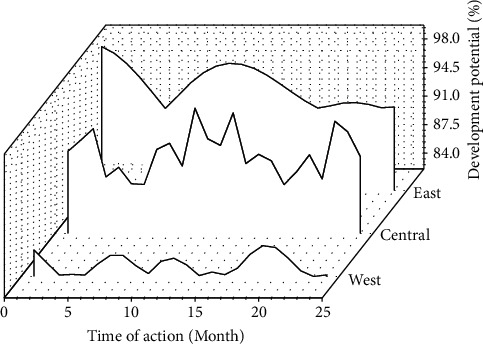
Regression results of the Hainan Free Trade Zone data by region.

The regression results are shown in Figure 3. Due to the differences in resources, history, and enjoyment policies in different regions, in order to further verify the effect of the establishment of the Hainan Free Trade Zone on different regions, the sample is based on the private fixed asset investment data released by the National Bureau of Statistics in 2019. The division rules are divided and brought into the model for testing. The results are shown in Figure 4.

**Figure 5:**
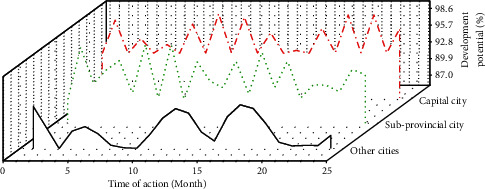
The data of the Hainan Free Trade Bonded Zone returns by city level.

Different levels of cities enjoy different policies and public resources, and accepting the same policy shock may have different effects. Therefore, the provincial capitals, subprovincial cities, and other cities in the sample cities are regressed separately. The regression of Hainan Free Trade Zone data by city level is shown in Figure 5.

### 4.3. Parallel Trend Test

The empirical research double-difference method used in this paper relies on the important premise that the experimental group and the control group meet the parallel trend; that is, if there is no impact of policies such as “setting up various Hainan Free Trade Bonded Zones or Free Trade Zones,” the trend should be consistent with that of the observation subjects in the control group. Therefore, the GDP data of each experimental group and the control group cities in the four years before the establishment of the first national-level Hainan Free Trade Zone were taken and the logarithm was used for observation.

**Figure 6:**
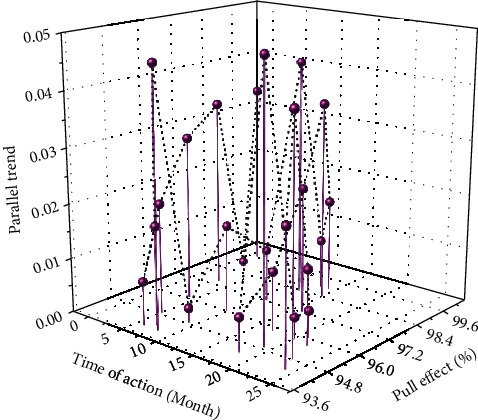
Parallel scope inspection of Hainan Free Trade Port.

By comparing the log (GDP) trends of the experimental group and the control group, it can be found that the situation before the implementation of the policy is exactly the same; that is, it satisfies the parallel trend test. After taking the logarithm, a parallel trend test is performed, and Figure 6 is obtained. From the comparison of data trends, it can be seen that the parallel range test of Hainan Free Trade Port satisfies the parallel trend test.

### 4.4. Replacing the Explained Variable Test

**Figure 7:**
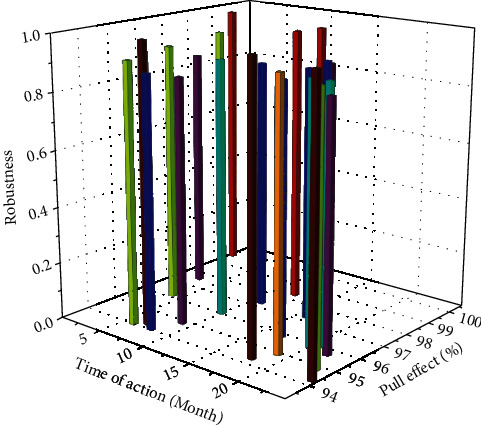
Robustness test of Hainan Free Trade Zone's economic boosting effect.

In order to further test the robustness of this result, the explained variable was replaced, and the logarithm of GDP per capita was taken as the explained variable to conduct a preliminary robustness test. Explanatory variables and other control variables remain unchanged. The results of the robustness test of the Hainan Free Trade Zone's effect on economic growth are shown in Figure 7.

When the explanatory variable is changed to the logarithm of per capita GDP, the sign of the coefficient of the explanatory variable of the Hainan Free Trade Zone group is still positive, and the policy of establishing the Hainan Free Trade Zone still has a significant effect on the regional economy. The Hainan Free Trade Port results are still not significant. By comparing the results of the two regressions, we can see that the two regressions of the two groups of data not only have roughly the same results but also the explanatory variable coefficients and *t* values are relatively close. This shows that the results of the double-difference are more credible. The establishment of the Hainan Free Trade Zone has had a significant effect on regional economic development, while the Free Trade Zone is still insignificant.

### 4.5. Testing considering the Lagging Effect of Policy

**Figure 8:**
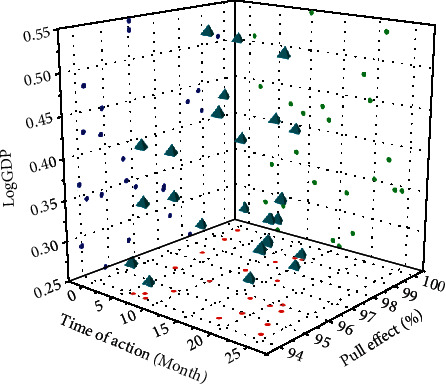
Results of the Hainan Free Trade Zone data after one period of lag.

In reality, there is often a time lag from the formulation to the release of the policy to the actual entry into force. Considering the impact of the establishment of the Hainan Free Trade Zone or Free Trade Zone and other preferential policies matching these two special economic zones on the economy, they do not necessarily act on the current period. Now, the explanatory variables are lagging by one period. It is assumed that the effects of these policies occur one year after the actual implementation year, and the double-difference model is used again to verify the net effect of the establishment of the Hainan Free Trade Zone policy on regional economic development. The explanatory variable is Log GDP.  Figure 8 shows the results of the Hainan Free Trade Zone data after one period of lag.

### 4.6. Contrastive One-Difference Test

At the beginning of the empirical part, when analyzing the reasons for choosing the difference-in-difference model, it was mentioned that the difference-in-difference method can more accurately measure the effect of policies. This is because the single-difference method lacks control variables, and the effects of other factors may also be classified as explanatory variables, resulting in overestimation of coefficients and exaggeration of significance. In order to verify whether this assumption is rigorous, the difference results are shown in Table 2.

By comparing the coefficients of the single-difference method and the double-difference method, it can be seen that the coefficient of the single-difference method is much higher than the estimation of the coefficient under the double-difference method. Then, it changes from insignificant to significant, and the sign of the coefficient changes from negative to positive. It can be seen that the estimation result of the single-difference method will indeed lead to overestimation. The fitting effect of the model under the estimation of the single-difference method is very poor. In summary, the results obtained by using the double-difference method are more accurate.

## 5. Conclusion

In view of the development of the Internet of Things industry chain, at this stage, the government should continue to increase the construction of information infrastructure and promote the balanced development of informatization. This task can only be solved by the central government's macrocontrol. If the information infrastructure is not complete, then everything is empty talk. Due to the different levels and speeds of economic development in different provinces, it is necessary to carry out interprovincial cooperation and communication in the IoT industry. This can not only gradually balance the development level of various provinces in China but also provide a platform for the unification of industry standards for the Internet of Things. From a policy perspective, the introduction of the IoT industry chain policy is still a framework-based requirement standard. Specific to the implementation process, various agencies and departments will not be able to coordinate and link to maximize effectiveness. To this end, a comprehensive coordination department should be launched to break down the barriers of interdepartmental communication and collaboration. Simple quantitative financial indicators to evaluate the profitability of enterprises can no longer meet the rapidly changing era of the economic environment and information technology. Multilevel, multiangle, and multifield comprehensive qualitative and quantitative indicators can be investigated in order to more accurately reflect the trend of continuous changes in profit models. It is not only difficult to control the impact of changing influencing factors on the profit model but also it is difficult to maintain a single profit model to meet the various profit needs of the enterprise. A suitable profit model to cope with the increasingly fierce market competition is the correct way to promote the development of enterprises. In order to more objectively verify the role of the Hainan Free Trade Zone and the Free Trade Zone on the economic development of the region, this paper adopts the double-difference method to test two different sets of data from the Free Trade Zone and the Hainan Free Trade Zone.

## Figures and Tables

**Table 1:** Descriptive statistics of the Hainan Free Trade Port data.

Variable name	Maximum value	Minimum value	Standard deviation
GDP per capita logarithmic	1.9	0.8	0.62
Budgetary expenditure	755.4	123.5	488.6
Natural population growth rate	54	35	6.7
Fixed asset investment	8775.7	50.8	2335.5
Gross domestic product logarithmic value	4.7	1.2	1.9
The output value of the secondary industry/gross domestic product in the current year	83.4	8.2	11.5

**Table 2:** Verification of robustness by comparing the single-difference method.

	Single-difference method	Double-difference method
Secondary industry as a share of GDP	0.89	0.93
Annual natural population growth rate	0.04	0.06
City/region individual fixed effects	Control	Control
Fixed asset investment	0.03	0.05
Whether to establish a bonded zone or a free trade zone	Yes	Yes
Expenditures in the budget	2.3%	2.4%

## Data Availability

The data used to support the findings of this study are available from the corresponding author upon request.
